# *CD58* polymorphisms associated with the risk of neuromyelitis optica in a Korean population

**DOI:** 10.1186/1471-2377-14-57

**Published:** 2014-03-24

**Authors:** Jason Yongha Kim, Joon Seol Bae, Ho Jin Kim, Hyoung Doo Shin

**Affiliations:** 1 Department of Life Science, Sogang University, 1 Shinsu-dong, Seoul 121-742, Republic of Korea; 2 Laboratory of Translational Genomics, Samsung Genome Institute, Samsung Medical Center, 81 Irwon-RoGangnam-Gu, Seoul 135-710, Republic of Korea; 3 Department of Neurology, National Cancer Center, 809 Madu 1-dong, Ilsandong-gu, Gyeonggi-do 410-769, Korea; 4 Department of Genetic Epidemiology, SNP Genetics, Inc, Seoul, Republic of Korea

**Keywords:** CD58, NMO, SNP, Haplotype

## Abstract

**Background:**

Neuromyelitis optica (NMO) is a serious inflammatory demyelinating disease (IDD), characterized by the inflammation and demyelination of optic nerves and spinal cords, which subsequently leads to the loss of function. In a previous genome-wide association study, *cluster of differentiation 58* (*CD58*) region was found to be susceptible for the risk of multiple sclerosis (MS) in Caucasian, and the association between *CD58* variants and MS was replicated in Americans. However, no study has been conducted to explore the possible association between *CD58* and NMO yet. Thus, this study aimed to investigate the association of *CD58* polymorphisms with the risk of NMO in a Korean population.

**Methods:**

Using TaqMan assay, 6 single nucleotide polymorphisms (SNPs) were genotyped in 98 NMO patients and 237 normal controls (N = 336). Logistic regression analysis was conducted to find a possible association between *CD58* polymorphisms and NMO.

**Results:**

The analysis results showed that 6 variations (*rs2300747*, *rs1335532*, *rs12044852*, *rs1016140*, *CD58_ht1*, and *CD58_ht3*) showed significant associations (*P* = 0.002 ~ 0.008, *P*^*corr*^ = 0.01 ~ 0.04).

**Conclusion:**

The genetic variations in *CD58* may be associated with the susceptibility of NMO in a Korean population. Based on previous studies, we suspect that the A allele of *rs2300747* may decrease CD58 RNA expression, thus increasing NMO risk. Also, we deduced that the G allele of *rs1016140* caused an increase of T cell activity, which in turn eased the access of AQP4 antibody into central nervous system (CNS) and ultimately leading to NMO development.

## Background

Neuromyelitis optica (NMO), which belongs to inflammatory demyelinating diseases (IDDs), is caused by the demyelination of axons in optic nerves and spinal cords. Although NMO has similarities with multiple sclerosis (MS), previous studies reported that MS and NMO may have different etiology [1-3]. In MS, demyelination causes symptoms such as a loss of sensitivity, hypoesthesia, parenthesis, disturbance of vision such as double vision, and muscle weakness. On the other hand, a loss of vision and spinal cord function are the most significant symptoms in NMO [1,2]. It is also known that the prevalence of MS is higher in Caucasians than Asians (1 ~ 4/100,000 in Asian vs. 30 ~ 150/100,000 in Caucasian) [4,5]. Contrary to MS, the prevalence of NMO is higher in non-Caucasians including African, Hispanic, and Asian [6].

Previous studies have shown that IDDs are complex-trait diseases with both genetic and environmental factors. However, compared to MS, there have been far less studies on NMO. In our previous study, a genome-wide association study (GWAS) was conducted for NMO and MS, showing that the risk polymorphisms for NMO and MS were different from each other [7]. Another study conducted in our group has shown that SNPs in *cluster of differentiation 6* (*CD6)* and *tumor necrosis factor receptor superfamily member 1A* (*TNFRSF1A*) were associated with NMO, but not with MS [8]. These studies have shown that there are definite merits in conducting genetic association studies of NMO independently.

Cluster of Differentiation 58 (CD58), also known as lymphocyte function-associated antigen 3 (LFA-3), is one of cell adhesion molecules abundantly expressed on antigen presenting cells (APCs) [9]. Conjugation between CD58 and CD2 or LFA-2, expressed in T-cell, is crucial for T-cell activation [10]. Furthermore, the importance of T-cell in MS development is well documented in previous studies [11,12]. To date, several association studies were conducted between *CD58* and IDDs since the first GWAS identified the gene’s association with MS [13]. Follow-up studies have shown that *CD58* SNPs such as *rs2300747* and *rs12044852* were associated with MS risk [14-18]. However, no study has looked into the association between *CD58* and NMO. Therefore, in the present study, we have conducted association studies between *CD58* polymorphisms and NMO in a Korean population.

## Methods

### Subjects

For genotyping of *CD58* polymorphisms, 98 NMO patients and 237 controls were recruited. In order to study biologically homogenous population, all the patients showed both optic neuritis and longitudinally extensive transverse myelitis following the revised diagnostic criteria for NMO [19], and seropositive for aquaporin-4 antibody [20]. Anti-AQP4 antibodies were measured by using an enzyme-linked immunosorbent assay (ELISA) [21] and cell-based assay (CBA) with a commercial slide kit (Euroimmun, Luebeck, Germany) [22]. In addition, 237 healthy controls of Korean ethnicity were included (Age = 47.3 (38.0 - 60.0), Female/Male = 156/81) who did not have characteristics of inflammatory demyelinating diseases including NMO, classical MS, optic neuritis and transverse myelitis. The study protocol was approved by the Institutional Review Board of the National Cancer Center of Korea. We obtained agreement of each subject by written information before beginning the study. The information of the subjects for present study is summarized in Table 1. Detailed demographic and clinical characteristics of NMO patients were previously described elsewhere [23].

**Table 1:** Characteristics of study subjects

	**NMO**	**Control**
Number of subject	98	237
Sex (M/F)	10/88	81/156
Age (mean (min.-max.))	39.9 (11–67)	47.3 (38–60)
Onset age (mean ± Std)	33.5 ± 12.26	-
Duration (year, mean ± Std)	7.0 ± 4.42	-

NMO, neuromyelitis optica.

### Single nucleotide polymorphism (SNP) selection and genotyping

Six SNPs of *CD58* were selected based on following conditions: (1) linkage disequilibrium (LD, minor allele frequency (> 0.05)), (2) locations (SNPs in exons were preferred), (3) previously reported SNP, (4) amino acid changes (non-synonymous SNPs were preferred). Genotype data of Asian (Chinese and Japanese) population from database of International HapMap Project (http://hapmap.ncbi.nlm.nih.gov/) were used for selection. Then, the selected SNPs were genotyped in 99 NMO cases and 237 healthy controls using TaqMan assay on the ABI prism 7900HT sequence detection system (Applied Biosystems, USA). The TaqMan primer information was listed in Additional file 1: Table S1.

### Statistics

LD was obtained using the HaploView software (version 4.2) from the Broad Institute (http://www.broadinstitute.org/mpg/haploview), with examination of Lewontin’s D’ (|*D’*|) and the LD coefficient *r*^*2*^ between all pairs of bi-allelic loci [24]. P-values for Hardy-Weinberg equilibrium (HWE) were also calculated using the HaploView software. Haplotypes were first estimated using PHASE software [25], and then computed using Statistical Analysis System (SAS). Associations for NMO under logistic model were adjusted by age (continuous value) and sex (male = 0, female = 1) as covariates using SAS. In order to correct for the multiple testing error, the SNPSpD program (http://gump.qimr.edu.au/general/daleN/SNPSpD/) was used, with the correction number of 4.5055. An *in silico* analysis was conducted by using Pupasuite 3.1 (http://pupasuite.bioinfo.cipf.es/) [26].

## Results

**Figure 1:**
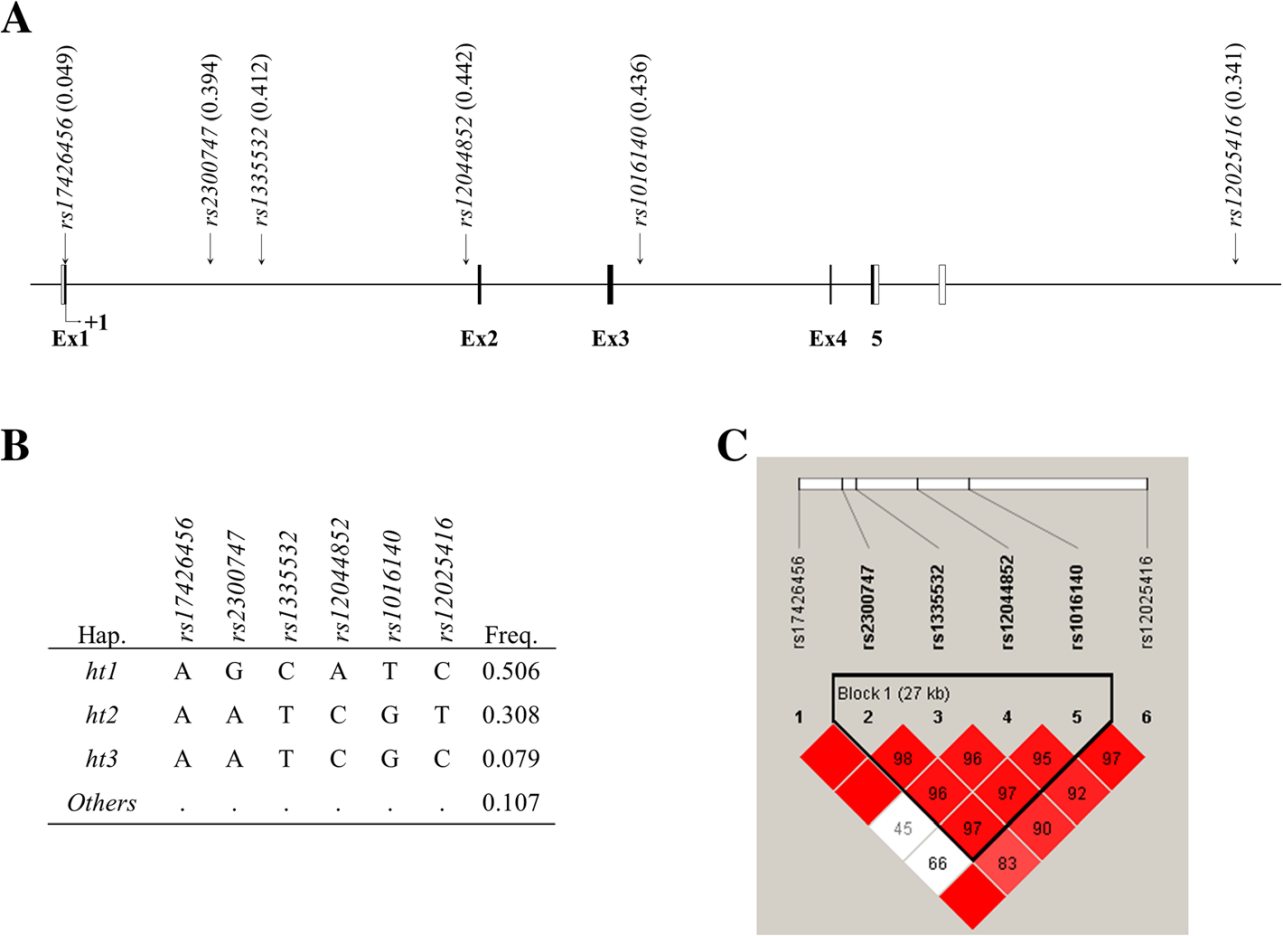
**Schematic physical map, haplotypes and LD status of *CD58 *polymorphisms. (A)** Polymorphisms identified in *CD58*. Coding exons are marked by shaded blocks and un-translated region (UTR) by white blocks. The LD coefficients (*r*^2^) are based on the genotypes of Korean samples. **(B)** Haplotypes of *CD58* in the Korean population. Only those with frequencies over 0.05 are shown. *Others* contain rare haplotypes: AATCGC, GGCATC, AGCCTC, AGTCGT, GGCCGC, AGCCGT, AGCAGT, AATATC, AGCATT, AGTCGC, AGTATC, AACCGC, AATAGT, and AACATC. **(C)** LD coefficients (|D’| and *r*^2^) among the selected SNPs based on the genotypes of whole study subjects in this study (n = 335).

In the present study, we obtained the genotype information of 336 subjects comprised of 98 NMO patients and 237 healthy subjects. Detailed clinical information about the subjects is summarized in Table 1. A total of 6 SNPs was genotyped for the study, and their location, haplotypes, and LD map are displayed in Figure 1. For the present study, only the haplotypes with frequencies over 0.05 were used. In addition, genotype frequencies, heterozygosity, and P-values of HWE are shown in Additional file 1: Table S2. All of the polymorphisms in the study were in HWE except *rs17426456*, which was located in the exonic region. The comparisons of *CD58* SNPs frequencies in Asian, Caucasian, and African populations showed that there were distinct differences among them (Additional file 1: Table S3). In order to compare the LD structures among populations, we have also drawn LD maps for African, Asian, and Caucasian in Additional file 1: Figure S1 (A, B, and C respectively). The results showed that the LD structure of Korean population was similar with that of Asian, but slightly different from that of Caucasian. In contrast, the LD structure of African population was clearly distinct from other populations.

In order to examine the possible association between *CD58* and NMO, we conducted association analysis between *CD58* polymorphisms and NMO (Table 2). Statistical analyses revealed that 4 SNPs (*rs2300747, rs1335532*, *rs12044852,* and *rs1016140*) and 2 haplotypes (*CD58_ht1* and *CD58_ht3*) showed significant associations with the risk of NMO (*P* = 0.002 ~ 0.008, *P*^*corr*^ = 0.01 ~ 0.04) (Table 2).

**Table 2:** **Association analysis using *CD58 *** **polymorphisms and haplotypes with NMO risk**

**SNP/Haplotype**	**MAF**		**OR (95% CI)**	***P***	***P***^***corr***^
	**Case**	**Control**			
	**(n = 98)**	**(n = 237)**			
*rs17426456*	0.046	0.051	0.92 (0.45-1.88)	0.79	NS
*rs2300747*	0.490	0.354	1.73 (1.23-2.43)	*0.007*	*0.03*
*rs1335532*	0.520	0.367	1.87 (1.33-2.63)	*0.002*	*0.01*
*rs12044852*	0.454	0.601	1.80 (1.28-2.53)	*0.004*	*0.02*
*rs1016140*	0.535	0.395	1.76 (1.25-2.47)	*0.005*	*0.02*
*rs12025416*	0.412	0.312	1.55 (1.09-2.20)	0.06	NS
*CD58_ht1*	0.456	0.451	1.69 (1.22-2.34)	*0.006*	*0.03*
*CD58_ht2*	0.234	0.281	1.52 (1.06-2.17)	0.12	NS
*CD58_ht3*	0.089	0.063	2.13 (1.19-3.84)	*0.008*	*0.04*

Logistic regression analyses were performed for calculating odds ratio (95% confidential interval) and *P*-values for SNP sites and haplotypes. Age (continuous value) and sex (male = 0, female = 1) were adjusted by inclusion in logistic analysis as covariates. To obtain the optimal correction for multiple testing of single-nucleotide polymorphisms (SNPs) in linkage disequilibrium (LD) with each other, the effective number of independent marker loci (4.5055) in *CD58* was calculated using the web based software SNPSpD (Http://genepi.qimr.edu.au/general/daleN/SNPSpD), on the basis of the spectral decomposition (SpD) of matrices of pairwise LD between SNPs. Significant associations (<0.05) are italicized. MAF, minor allele frequency; *P*^*corr*^, corrected *P*-value using multiple testing corrections; OR, odds ratio; CI, confidence interval; NMO, neuromyelitis optica.

## Discussion

In the present study, we have conducted logistic analysis to find a possible significant association between *CD58* polymorphisms and NMO in a Korean population. Previously, several studies have reported the associations of *CD58* polymorphisms such as *rs12044857*, *rs1335532* and *rs2300747,* with MS [13,17,18]. Our results showed that the SNPs reported to be associated with MS (*rs2300747*, *rs13355332*, and *rs12044852)* in previous studies [15,17] were significantly associated with NMO as well. In addition, *rs1016140*, which was not previously studied for MS or NMO, also showed a significant association in our results.

Although there exist some differences between MS and NMO etiologies [27], the two diseases still share similar symptoms and onset mechanisms, in which the body’s immune systems are misdirected to attack its own CNS. While *CD58* polymorphisms had never been studied in association with NMO, there were several studies which reported significant associations of *CD58* variants with MS. Therefore, in Table 3, we listed the results of such studies and compared them with the present study result. The comparison showed that while the diseases and ethnicities were different between the previous MS studies and the present NMO study, the polymorphisms *rs2300747* (OR = 1.20 - 1.39 in previous MS studies and 1.80 in the present study, P <0.05 in all studies), *rs1335532* (1.28 in a previous MS study with Caucasian populations and 1.87 in the present study, P < 0.05 in both studies), and *rs12044852* (1.22 and 1.56 in previous MS studies (P = 1.1 × 10^−6^ and 0.093 respectively) and 1.73 in the present study (P = 0.007)) were significantly associated with both MS and NMO in similar trends.

**Table 3:** **Comparison of previous studies on *CD58 *– MS/NMO association**

**Reference**	**Study populations**	**Study subjects**	**Study allele**		
		**(case/control)**	***rs12044852***	***rs1335532***	***rs2300747***
			***P-value *** **(OR)**	***P-value *** **(OR)**	***P-value *** **(OR)**
Hafler et al. (2007) [13]	US and UK (MS)	2322/5418 (1540 family trios)	*1.9 × 10*^*−5*^ (1.24)	-	-
Rubio et al. (2008) [16]	Australia (MS)	1134/1265	*0.042* (1.20)	-	-
Bahlo et al. (2009) [18]*	Australia, NZ, UK, and US (MS)	3874/5723	-	*9.6 × 10*^*−8*^ (1.28)	-
De Jager et al. (2009) [17]*	US, UK, Belgium, Japanese, Chinese, and Finland (MS)	3558/4420 (1768 family trios)	-	-	*1.1 × 10*^*−6*^ (1.22)
Brynedal et al. (2009) [28]*	Swedish (MS)	1077/1217	*4.3 × 10*^*−4*^ (1.39)	-	**-**
Qiu et al. (2013) [29]	Australia (MS)	350/498	-	-	0.093 (1.56)
Present study	Korean (NMO)	99/237	*0.004* (1.80)	*0.002* (1.87)	*0.007* (1.73)

*Since these studies had reversed minor and major alleles compared to the present study, we modified their results to conform to our results. Italicized *P*-values are values below 0.05, which indicate significance.

In order to further study the function of the *CD58* SNPs, we have conducted *in silico* analysis of the 4 intronic SNPs associated with NMO. As a result, no SNPs were predicted to cause alternative splicing or be an exonic splicing enhancer or silencer (data not shown). However, previous reports suggest that there may be functional backgrounds on at least 2 SNPs, *rs2300747* and *rs1016140*. In a recent study, the G allele of *rs2300747* was found to limit the MS inflammation by increasing the *CD58* RNA expression [17]. In our study, we showed that the A allele of *rs2300747* was associated with the increased risk for NMO (P_cor._ = 0.03, OR (95% CI) = 1.73 (1.23-2.43)). We suspect that the decreased *CD58* RNA expression, caused by the A allele, may increase NMO risk as well, although further functional studies are required to confirm this notion.

A recent study has shown that T cell-mediated central nervous system (CNS) inflammation is a pre-requisite for the access of AQP4 antibody into CNS, an integral step in the development of NMO [30]. In a separate study, it was reported that *rs1016140* was associated with the decreased peak antibody level of hepatitis B virus and decreased T cell activity [31]. Another study also reported that the *CD58* haplotype (*rs1414275*-*rs11588376*-*rs1016140*) might affect the response level of CD58 with T cell co-stimulatory molecules to decrease the T cell activity [32]. In the present study, the G allele of *rs1016140* was associated with the increased NMO risk. We suspect that the increased T cell activity caused by the G allele may lead to the more robust CNS inflammation, which in turn eases the access of AQP4 antibody into CNS, and ultimately leads to NMO development. Further studies would be needed to confirm our notion about the role of *rs1016140* in the NMO etiology.

Although our study reports a potential association between *CD58* polymorphisms and NMO, some limitations are present which should be addressed in the future. First, number of patients and controls enrolled in the study was relatively small, due to the rarity of the disease. This might have caused the low P-value of HWE for *rs17426456*. Second, there was a disparity in the gender ratio, as there were far more female subjects than male subjects in the study. However, it has been reported that NMO is approximately 3 to 5 times more common in women than men [33]. In addition, association analysis was adjusted for gender to accommodate for this disparity. Lastly, functional study would be required to examine the actual effect of *CD58* SNPs.

## Conclusions

We have conducted association analyses between *CD58* polymorphisms and NMO to find that 4 SNPs and 2 Haplotypes of *CD58* were significantly associated with the increased risk of NMO. From previous studies, we have deduced the possible functional background of *rs2300747* and *rs1016140*, but the roles of other polymorphisms remain unknown. Our study is the first to find significant association between *CD58* polymorphisms and NMO in a Korean population. However, further studies may be required to confirm the functional role of *CD58* polymorphisms with NMO. We expect the results in the present study to provide a new insight on the role of *CD58* in NMO and be helpful in developing new treatments for the diseases.

## Consent

Written informed consent was obtained from the patient for the publication of this report and any accompanying images.

## Competing interests

The authors declare that we have no competing interest.

## Authors’ contributions

JYK and JSB analyzed the data and wrote the manuscript. HJK supplied the raw data. HJK and HDS conceived the study. All authors read and approved the final manuscript.

## Authors’ information

Jason Yongha Kim and Joon Seol Bae are joint first authors.

## Pre-publication history

The pre-publication history for this paper can be accessed here:

http://www.biomedcentral.com/1471-2377/14/57/prepub

## Supplementary Material

Additional file 1: Table S1 Primer/probe information of CD58 SNPs. **Table S2.** Genotype frequencies of CD58 and P-value of deviations of Hardy-Weinberg equilibrium in a Korean population. **Table S3.** Minor allele frequencies of selected CD58 polymorphisms using data from HapMap project. **Figure S1.** Linkage disequilibrium plots for selected CD58 polymorphisms in different races. LD plots were based on data from International HapMap Project. (A) LD plot of African. (B) LD plot of Asian. (C) LD plot of Caucasian. Click here for file
