# Supplementary material for: CD58 polymorphisms associated with the risk of neuromyelitis optica in a Korean population
Source: BMC Neurol. 2014 Mar 24;14:57. doi: 10.1186/1471-2377-14-57 (PMC3998011; doi:10.1186/1471-2377-14-57)

**Additional file**

Supplementary Figure 1. Linkage disequilibrium plots for selected *CD58* polymorphisms in different races. LD plots were based on data from International HapMap Project. (A) LD plot of African. (B) LD plot of Asian. (C) LD plot of Caucasian.

Table 1. Primer/probe information of *CD58* SNPs

| Loci |  | Primer/probe sequence or Assay-by-design ID^*^ | Methods |
| --- | --- | --- | --- |
| *rs17426456* | Forward | GAACTTAGGGCTGCTTGTGG | TaqMan assay by design |
|  | Reverse | CGTCTCTGATCGGCAACC |  |
|  | VIC | GTCCTCAGCGTG |  |
|  | FAM | GTCCTCGGCGTG |  |
| *rs2300747* |  | C__15755405_10 | TaqMan assay |
| *rs1335532* |  | C___8700717_10 |  |
| *rs12044852* |  | C__31433800_10 |  |
| *rs1016140* |  | C__26629015_10 |  |
| *rs12025416* |  | C__31433762_10 |  |

^*^TaqMan assay IDs from Applied Biosystems, Foster City, CA, USA.

Table S2. Genotype frequencies of *CD58* and *P*-value of deviations of Hardy-Weinberg equilibrium in a Korean population

| Loci | Location | Allele | Hetero- |  | Genotype | | |  | HWE |
| --- | --- | --- | --- | --- | --- | --- | --- | --- | --- |
|  |  | change | zygosity |  | C/C | C/R | R/R |  |  |
| *rs17426456* | Exon1 | A>G | 0.094 | Total | 305 | 25 | 4 |  | 0.0002 |
|  |  |  |  | Case | 89 | 7 | 1 |  | 0.069 |
|  |  |  |  | Control | 216 | 18 | 3 |  | 0.001 |
| *rs2300747* | Intron1 | G>A | 0.477 | Total | 126 | 155 | 54 |  | 0.583 |
|  |  |  |  | Case | 27 | 47 | 24 |  | 0.693 |
|  |  |  |  | Control | 99 | 108 | 30 |  | 0.948 |
| *rs1335532* | Intron1 | C>T | 0.484 | Total | 118 | 159 | 58 |  | 0.724 |
|  |  |  |  | Case | 22 | 51 | 25 |  | 0.679 |
|  |  |  |  | Control | 96 | 108 | 33 |  | 0.766 |
| *rs12044852* | Intron1 | A>C | 0.493 | Total | 107 | 159 | 67 |  | 0.571 |
|  |  |  |  | Case | 21 | 46 | 29 |  | 0.732 |
|  |  |  |  | Control | 86 | 113 | 38 |  | 0.931 |
| *rs1016140* | Intron3 | T>G | 0.491 | Total | 109 | 161 | 65 |  | 0.688 |
|  |  |  |  | Case | 21 | 50 | 27 |  | 0.810 |
|  |  |  |  | Control | 88 | 111 | 38 |  | 0.762 |
| *rs12025416* | 3'-UTR | C>T | 0.448 | Total | 145 | 150 | 38 |  | 0.933 |
|  |  |  |  | Case | 30 | 54 | 12 |  | 0.104 |
|  |  |  |  | Control | 115 | 96 | 26 |  | 0.381 |

C/C, C/R, and R/R indicate the homozygotes for the common allele, and heterozygotes and homozygotes for the rare allele, respectively. HWE, *P*-values of deviation of Hardy-Weinberg equilibrium.

Table S3. Minor allele frequencies of selected *CD58* polymorphisms using data from HapMap project.

|  | Race | N | *rs2300747* | *rs1335532* | *rs12044852* | *rs1016140* | *rs12025416* |
| --- | --- | --- | --- | --- | --- | --- | --- |
|  |  |  | G>A | C>T | A>C | T>G | C>T |
| MAF | Caucasian | 174 | 0.135 | 0.138 | 0.131 | 0.135 | 0.198 |
|  | Asian | 590 | 0.378 | 0.393 | 0.419 | 0.416 | 0.320 |
|  | African | 176 | 0.222 | 0.425 | 0.069 | 0.194 | 0.234 |

Minor allele frequencies were calculated based on information from International HapMap Project. African includes YRI (Yoruban in Ibadan, Nigeria). Asian include CHB (Han Chinese in Beijing, China), JPT (Japanese in Tokyo, Japan) and Korean from the present manuscript. Caucasian includes CEU (Utah residents with Northern). MAF, minor allele frequency.

Figure S1.


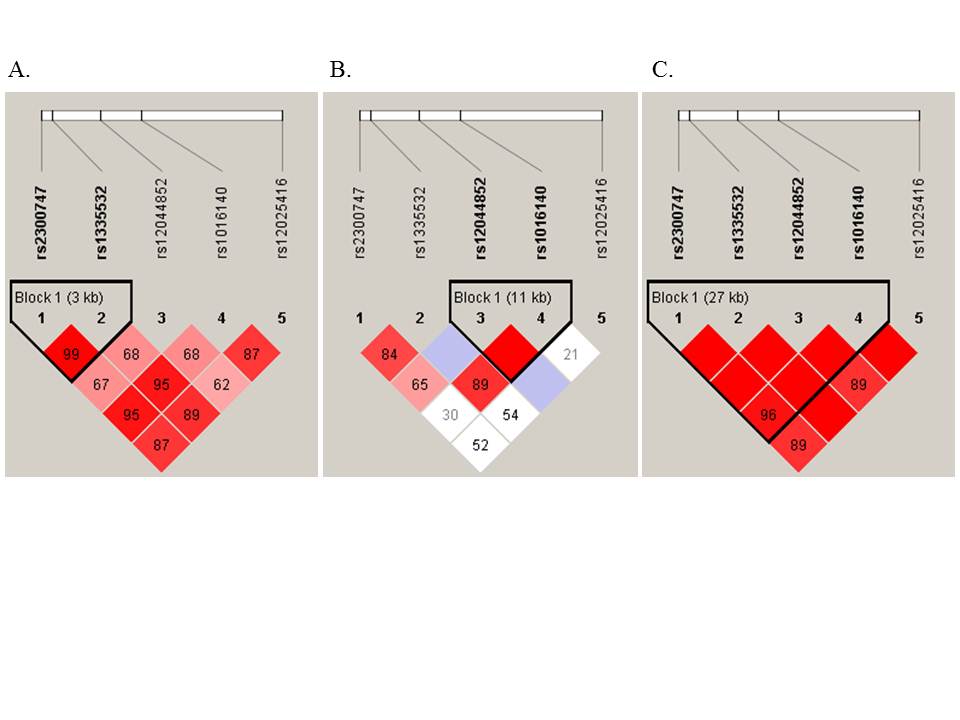

Supplement: Additional file 1: Table S1 — Primer/probe information of CD58 SNPs. Table S2. Genotype frequencies of CD58 and P-value of deviations of Hardy-Weinberg equilibrium in a Korean population. Table S3. Minor allele frequencies of selected CD58 polymorphisms using data from HapMap project. Figure S1. Linkage disequilibrium plots for selected CD58 polymorphisms in different races. LD plots were based on data from International HapMap Project. (A) LD plot of African. (B) LD plot of Asian. (C) LD plot of Caucasian. [file 1471-2377-14-57-S1.doc]
